# Targeted Drug Delivery of Microbubble to Arrest Abdominal Aortic Aneurysm Development: A Simulation Study Towards Optimized Microbubble Design

**DOI:** 10.1038/s41598-020-62410-3

**Published:** 2020-03-25

**Authors:** Amir Shamloo, Sina Ebrahimi, Ali Amani, Famida Fallah

**Affiliations:** School of Mechanical Engineering, Sharif University of Technology, Tehran, Iran

**Keywords:** Biomedical engineering, Mechanical engineering, Biophysics, Biotechnology, Computational models

## Abstract

Abdominal aortic aneurysm (AAA) is an irreversible bulge in the artery with higher prevalence among the elderlies. Increase of the aneurysm diameter by time is a fatal phenomenon which will lead to its sidewall rupture. Invasive surgical treatments are vital in preventing from AAA development. These approaches however have considerable side effects. Targeted drug delivery using microbubbles (MBs) has been recently employed to suppress the AAA growth. The present study is aimed to investigate the surface adhesion of different types of drug-containing MBs to the inner wall of AAA through ligand-receptor binding, using fluid-structure interaction (FSI) simulation by using a patient CT-scan images of the vascular system. The effect of blood flow through AAA on MBs delivery to the intended surface was also addressed. For this purpose, the adherence of four types of MBs with three different diameters to the inner surface wall of AAA was studied in a patient with 40-mm diameter aneurysm. The effects of the blood mechanical properties on the hematocrit (Hct) percentage of patients suffering from anemia and diabetes were studied. Moreover, the impact of variations in the artery inlet velocity on blood flow was addressed. Simulation results demonstrated the dependency of the surface density of MBs (SDM) adhered on the AAA lumen to the size and the type of MBs. It was observed that the amount of SDM due to adhesion on the AAA lumen for one of the commercially-approved MBs (Optison) with a diameter of 4.5 μm was higher than the other MBs. Furthermore, we have shown that the targeted drug delivery to the AAA lumen is more favorable in healthy individuals (45% Hct) compared to the patients with diabetes and anemia. Also, it was found that the targeted drug delivery method using MBs on the patients having AAA with complicated aneurysm shape and negative inlet blood flow velocity can be severely affected.

## Introduction

Abdominal aortic aneurysm (AAA) is one of the most common types of cardiovascular diseases (CVDs) occurring in the middle part of the aorta. AAA is originated from the changes in the mechanical properties of the vessel wall leading to permanent focal dilation of 50%^1^. At present, small AAAs, with a diameter of less than 55 mm in men and less than 50 mm in women, are examined with regular monitoring in terms of size. At this point, the growth rate is approximately 3–10 mm/year. The patient needs surgery in case of AAA excessive enlargement beyond these values^2–4^. However, the minimally invasive or so-called endovascular method also has some complications, including difficulty and complexity of the operation when AAA is closed to one of the visceral veins. Also, about 20 percent of grafts cannot completely remove the damaged vessel from the circulatory system^5,6^. Another problem with the endovascular method is the durability of this method due to endoleak (continuous blood leakage around stents), which results in shorter durability of this type of treatment^5^. Despite these drawbacks and challenges, effective treatment is still needed to reduce or hinder the aneurysm growth The use of the drug for AAA treatment is one efficient method with negligible side effects which has become popular in recent years^7–9^.

Clinical studies have shown that doxycycline, estrogen, renin-angiotensin system inhibitors, statin and β-receptor angiotensin have high potential in preventing the AAA growth^9,10^. Recently, it has been demonstrated that the Cycloastragenol (CAG) not only can hinder the aneurysm growth, but it can also reduce the AAA diameter^8^. Drug injection is generally used in AAA. This treatment requires direct contact of the drug particles with the inner wall of the AAA to hinder or prevent the aneurysm growth^11^. AAAs have various complicated shapes. Consequently, the blood flow field will have different velocities and pressures which may hinder the drug delivery to the artery. This can lead to a significant reduction in the efficacy of the drug delivery method. In this regard, the targeted drug delivery method could play a significant role^11–14^.

Currently, micro- and nanoparticles have drawn a considerable deal of attention in the field of the targeted drug delivery^15–17^. In this content, particles with optimised diameter and the shape have been designed to increase the adhesion and the migration ability to the target site^18,19^. Moreover, studies have shown that the geometry of the vessel, velocity and pressure of blood are the key parameters in optimisation of the particles shape and size. Also, it can affect the migration and adhesion of particles to the targeted surface^12^. One possible method to enhance the effectiveness of drug delivery is use of microbubbles (MBs) and ultrasound^11,20,21^. MBs used in targeted drug delivery can vary according to the vessel type. MBs shell material, core gas, MBs production and ultrasound method, materials and targeting ligands are among the parameters with decisive role in the targeted drug delivery success^22–24^. Generally, clinically and experimentally approved MBs consist of two parts. A core including the perfluorocarbons and stabiliser membrane which is made from different materials like polymers, phospholipids and proteins. The diameter of the produced MBs varies based on their species; but particles with diameter range of 1–5 *μm* are generally used in pharmacy. In some cases, application of MBs with diameter of 10 *μm* was reported as well^25–27^.

First, the design of drug-loaded MBs targeting the cardiovascular tissues received the attention of the researchers. Then, liposome was investigated as a powerful tool in targeted drug delivery^28,29^. After a description of MBs designs, researchers examined the drug carriers targeting the endothelial cells^23,30,31^. Regarding the studies of targeted drug delivery in CVDs, numerous factors can hinder drug diffusion in the artery lumen. Each effect has been investigated in the large arteries^32^. A huge deal of studies also addressed the targeted drug delivery using drug-carrying particles for coronary arteries^12,13,33^. However, no study was found concerning the effect of MBs size and type (two factors with significant roles in adhesion to the artery wall). Optimisation of MBs size for efficient adhesion to the wall, ease of construction and economic aspects are of great importance.

In this study, fluid-structure interaction (FSI) simulations were performed based on patient-specific geometry in which an incompressible and isotropic arterial wall behavior, non-Newtonian blood behavior and physiological boundary conditions are considered. Then, targeted drug delivery by MBs has been investigated in AAA. Among the commercially-approved MBs, Definity, Micromarker, Optison and Sonovue were used for simulation. MBs size was studied as a function of surface adhesion and other important factors. The effects of the blood mechanical properties and the velocity of the blood flow through the artery on the surface adhesion (which influences AAA) were also investigated. Besides, the surface density of MBs (SDM) on lumen AAA, average MBs residence time (MRT) and the effects of forces exerted on MBs on delivery to the targeted surface were examined.

## Materials and Methods

In this section, first, the methods used for determination of the patient-specific geometry and the mechanical properties of the blood and the artery wall are described. Then, the mesh study and solution dependence on time were addressed. The boundary condition of the simulation and FSI formulation are also expressed. The mechanical properties of each MB are also provided. Particle tracking for fluid flow was explained for MBs motion within the artery. The force applied by blood flow to the MBs as well as the MB-MB forces is formulated. Finally, MB adhesive dynamic model was briefly described based on the MBs adhesion to the target through the ligand-receptor binding.

### Geometry reconstruction

**Figure 1:**
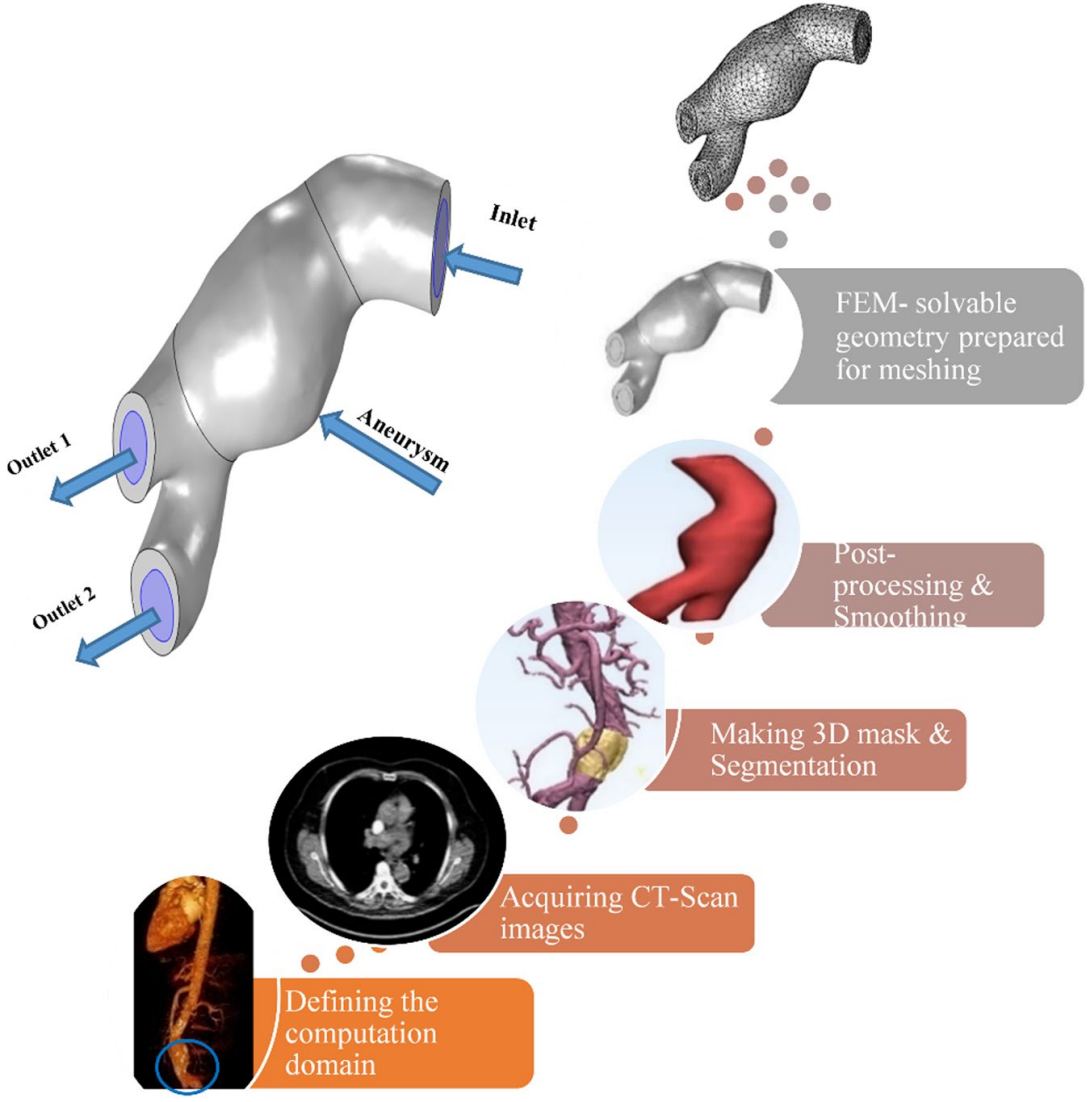
Steps taken to obtain the FEM solvable geometry of the AAA (Generated partly by COMSOL Multiphysics 5.3, https://www.comsol.com).

It has been demonstrated that the local fluid flow velocity in AAA depends strongly on the shape of AAA; thus, the AAA geometry affects the transition of MBs and their deposition^34,35^. Therefore, in this study, patient-specific geometry has been used to reach accurate results. The CT-scan images (DICOM format) of a 75-year-old male patient was acquired from Tehran Heart Center, Tehran, Iran. This was carried out in accordance with relevant hospital guidelines and regulations which was approved by Tehran Heart Center hospital and the informed consent was obtained from the patient. An image-processing software was utilised to reconstruct a 3D model from 2D images. The model was manually modified to remove Additional branches. Figure 1 shows the process of geometry reconstruction for finite element meshing. Also, the different segments of the geometry are shown in this figure. AAA segment (an irreversible bulge) is the part which is bigger in diameter from the normal condition. For this patient, the maximum aneurysm diameter occurs approximately in the middle part with a magnitude of 40 mm. The aneurysm has a non-uniform thickness with an average of 4 mm. The inlet lumen area of the abdominal aorta was 377.4 mm^2^, and the lumen areas at the bifurcation were 189.7 and 163.8 mm^2^.

### Arterial wall and blood properties

The isotropic Raghavan *et al*. strain energy density model has been used for describe the mechanical properties of the arterial wall. The strain energy density function is as follows^36^:

1 $${W}_{iso}={C}_{10}({I}_{1}\,-3)+{C}_{20}{({I}_{1}-3)}^{2}+\frac{{(J-1)}^{2}}{d}$$

where ${W}_{iso}$ is the strain energy density of the material, ${C}_{10}$ and ${C}_{20}$ are the material constants, ${I}_{1}$ is the first deviatoric strain invariant, $J$ is the ratio of the deformed elastic volume over the undeformed volume materials and $d$ is the material incompressibility parameter. The AAA wall mechanical properties have been shown in Table 1.

**Table 1:** Material properties for AAA wall^71^.

Hyperelastic model	*C*_20_[*MPa*]	*C*_20_[*MPa*]	*d* [Dimensionless]	*ρ*[*kg*/*m*^3^]
AAA	0.174	1.881	1.149	1120

Many parameters determine the blood mechanical properties in the body. Previous studies show that Anemia, Diabetes, hypertension and spasm might alter the blood concentration^37,38^. These changes are associated with blood viscosity where hematocrit (Hct) is a primary factor^39^. Here, the Carreau-Yasuda model is implemented to describe the properties of the blood^40^. The shear rate-dependent non-Newtonian apparent blood dynamic viscosity was defined as follows^40^:

2 $${\eta }_{app}={\eta }_{\infty }+({\eta }_{0}-{\eta }_{\infty }){[1+{(\lambda \dot{\gamma })}^{2}]}^{(a-1)/2}$$

where ${\eta }_{\infty }$ and ${\eta }_{0}$ are the viscosity of fluid with infinite shear and the viscosity of fluid without shear, respectively. $\lambda$ represents the time constant, $\dot{\gamma }$ is the local shear rate and $\,a$ is the power-law index. The numerical values of parameters in Eq. (2) have been shown in Table 2.

**Table 2:** Carreau–Yasuda model values for the related parameters^40^.

Hematocrit percentage	*η*_0_ [*Pa*.*s*]	*λ*[*s*]	*a*[Dimensionless]	*η*_*∞*_ [*Pa*.*s*]
25%	0.0178	12.448	0.330	0.00257
45%	0.161	39.418	0.479	0.00345
65%	0.8592	103.088	0.389	0.00802

Supplemental Fig. S1 shows the blood fluid viscosity versus cardiac cycle for negative and non-negative inlet velocity. It is seen that for the negative inlet velocity, maximum viscosity (28.117 $mPa\cdot s$) occurs at $t=2.77\,s$ and for non-negative inlet velocity, it occurs at $t=3.14\,s$ and is equal to $96.163\,mPa\cdot s$.

### Mesh study and solution dependence on time

Arbitrary Lagrangian-Eulerian (ALE) method was used to combine solid mechanics using a Lagrangian description and a material frame with the fluid formulated using Eulerian description and a spatial frame. Additionally, the Navier-Stokes equations were chosen to describe the characteristics of the fluid flow. A non-uniform ALE moving mesh was generated for the entire geometry where mesh elements comprised of tetrahedral, prism, triangular, and quadrilateral elements. To investigate mesh independence, four different grids were selected to compare simulations results (Table 3). Considering both the accuracy and the computational time, 40771 elements was selected for all simulations presented in this study. Also, the time-independence of results has been assessed through studying FSI results variations in two cardiac cycles succeeding three cardiac cycles used in simulations (Table 4).

**Table 3:** Mesh refinement study in the FSI simulations.

Mesh elements	Max. velocity magnitude at the outlet 2 (m/s)	Avg. velocity magnitude (m/s)	Max. displacement of the arterial wall (mm)
33584	0.63122	0.073584	2.0558
37942	0.63689	0.075067	2.0517
^*^40771	0.62789	0.074415	2.0556
45736	0.62780	0.074041	2.0552
53576	0.63636	0.073584	2.0569

**Table 4:** Selected FSI results for five cardiac cycles.

Cycle number	Time interval (s)	Max. arterial wall displacement (mm)	Avg. velocity at the AAA outlet 2 (m/s)
1	0.6–1.6	2.0587	0.31255
2	1.6–2.6	2.0506	0.32848
3	2.6–3.6	2.0557	0.32877
4	3.6–4.6	2.0579	0.32845
5	4.6–5.6	2.0525	0.32784

### FSI and boundary conditions

Since the fluid has non-Newtonian properties, its motion can not be modeled with the Navier-Stokes equations. Generalised Newtonian Fluid (GNF)^41–44^ can model incompressible non-Newtonian fluids dynamics and is written similar to a Newtonian one, while considers the apparent dynamic viscosity instead of the dynamic viscosity as expressed in Eq. (2). This equation along with continuity equation is expressed as follows^43^:

3a $$\rho \frac{\partial {\bf{u}}}{\partial t}+\rho {\bf{u}}.\nabla {\bf{u}}=-\,\nabla p+\nabla \tau $$

3b $$\nabla .{\bf{u}}=0$$

where $\rho$, ${\bf{u}}$ and $p$ are the density, the velocity vector and the pressure of the fluid, respectively. Also, the stress tensor components ${\tau }_{ij}$ are proportional to the strain rate tensor components ${S}_{ij}$ as follows^41^:

4a $${\tau }_{ij}=2{\eta }_{app}{S}_{ij}-p{\delta }_{ij}$$

4b $${S}_{ij}=\frac{1}{2}\left(\frac{\partial {u}_{i}}{\partial {x}_{j}}+\frac{\partial {u}_{j}}{\partial {x}_{i}}\right)-\frac{1}{3}\frac{\partial {u}_{k}}{\partial {x}_{k}}{\delta }_{ij}$$

Here, ${\delta }_{ij}$ is the Kronecker delta function defined as follows:

5 $${\delta }_{ij}=\{\begin{array}{c}1\,if\,i=j\\ 0\,if\,i\ne j\end{array}$$

The blood fluid in the artery has an interaction with the wall of the artery, which causes force to the artery wall. The total force, ${\bf{f}}$, exerted on the solid boundary is as follows^13^:

6 $${\bf{f}}={\bf{n}}.\left[-p{\bf{I}}+{\eta }_{app}[(\nabla {\bf{u}}+{(\nabla {\bf{u}})}^{T})-\frac{2}{3}(\nabla .{\bf{u}}){\bf{I}})]\right]$$

where ${\bf{n}}$ is the outward normal vector to the boundar, ${\bf{I}}$ is the identity matrix, ${\eta }_{app}$ is the apparent dynamic viscosity of the fluid.

Equation (6) was solved based on the spatial coordinate while the solid domain was described in the material coordinate. Thus, the force needs to be changed as follows^13^:

7 $${\bf{F}}={\bf{f}}\frac{dv}{dV}$$

where $dV$ and $dV$ are the mesh element scale factors for the spatial frame and the material frame, respectively. Due to the lack of patient-specific data, the pulsatile velocity and pressure waveforms were considered as reported in^45^. In this study, a non-negative inlet velocity was also taken into account in order to investigate the effects of the velocity of the blood flow through artery on the SDM adhesion containing the drug. Supplemental Fig. S2 shows the negative inlet velocity, non-negative inlet velocity and pressure waveforms which were obtained for three cardiac cycles. The pulsatile pressure and velocity were applied to the inlet and outlets, respectively. The laminar flow was also assumed in this study.

### MBs and their properties

In the present study, four different types MBs were utilised to perform the targeted delivery in patients with AAA. Definity MBs usually consist of lipid sell, including phospholipids (DPPC, DPPA, MPEG5000DPPPE) which has an Octofluoropropane (C_3_F_8_) gas core. The Micromarker MBs have one layer of Polyethylene glycol, phospholipids and fatty acids in which there is a mixture of Nitrogen gas and Perfluorobutane gas (C_4_F_10_) inside the MBs^27,46^. Optison MBs consists of human serum albumin sell, which have a (C_3_F_8_) gas core. Finally, Sonovue MBs consists of lipid sell, including Zwitterionic phosphatidylcholine and an anionic phospholipid which have an (SF_6_) gas core^46–49^. In this study, a density of 1000 $Kg/{m}^{3}$ for MBs^50^ was considered. Moreover, the numerical values of MBs’ mechanical properties are presents in Table 5.

**Table 5:** Material properties for MBs.

Agent formulation	Shell	Gas	Diameter (${\boldsymbol{\mu }}{\boldsymbol{m}}$)	Surface tension $({\boldsymbol{N}}{\boldsymbol{/}}{\boldsymbol{m}})$	Dynamic viscosity $({\boldsymbol{P}}{\boldsymbol{a}}.{\boldsymbol{s}})$	Elasticity $({\boldsymbol{N}}{\boldsymbol{/}}{\boldsymbol{m}})$	Reference
Definity	Lipid	${C}_{3}{F}_{8}$	1.1–3.3	0.051	0.00415	0.855	^27,46^
Micromarker	Phospholipid	${C}_{4}{F}_{10}$	2.0–5.0	0.051	0.00952	5.150	^27,46^
Optison	Albumin	${C}_{3}{F}_{8}$	3.0–4.5	0.072	0.001	0.230	^46,47^
Sonovue	Phospholipid	$S{F}_{6}$	1.5–2.5	0.073	0.001	0.55	^48,49^

### Particle tracking for fluid flow

In the simulation performed in this study, in the total number of 2000 MBs with properties given in section 2.5 were released at the inlet section of the abdominal in four injections. The MBs were released at the onset of one-second intervals; times of injection were 0, 1, 2, 3 seconds during the total three cardiac cycles plus a ramp and entered randomly^12,13^.

In order to acquire realistic MB dynamics in blood flow, a mixed scattering diffusion method with the specular reflection method was adopted in the arterial wall-MBs interactions in order to simulate the influence of unsmooth surface of endothelial on the motion of MBs. The MB has probability γ = 0.5 to be reflected specularly. In specular reflection, the MB is reflected from the surface in the tangent plane. The incident angle and reflected angle are the same concerning the surface normal; hence, we have^13^:

8a $${\bf{q}}{\prime} ={\bf{q}}$$

8b $${\bf{v}}{\prime} ={\bf{v}}-2({\bf{n}}.{\bf{v}}){\bf{n}}$$

where ${\bf{q}}{\prime}$ and ${\bf{q}}$ are the MB position vector after and before contact with the surface, respectively. Likewise, ${\bf{v}}{\prime}$ is the post-contact particle velocity vector and ${\bf{v}}$ is precontact particle velocity vector. Also, ${\bf{n}}$ is the outward surface normal vector.

The particle is reflected diffusely; it is reflected from the surface with a velocity vector according to Knudsen’s cosine law. For the particle position, there is Eq. (8a), and for the particle velocity in 3D can be written^13^:

9a $${v}_{t1}=|{{\bf{v}}}_{c}|sin\,\theta \,\sin \,\varnothing $$

9b $${v}_{t2}=|{{\bf{v}}}_{c}|sin\,\theta \,\cos \,\varnothing $$

9c $${v}_{n}=|{{\bf{v}}}_{c}|\sin \,\theta $$

where ${{\bf{v}}}_{c}$ is the velocity vector when the particle strikes the surface, ${v}_{t1}$ and ${v}_{t2}$ are the outcoming-tangential components of the velocity, and ${v}_{n}$ is the normal component of the velocity. The angle $\varnothing$ is a uniformly distributed random number between 0 and 2π. Correspondingly, the angle $\theta$ has been defined as:

10 $$\theta ={\sin }^{-1}(\sqrt{\varGamma })$$

where $\varGamma$ is a uniformly distributed random number between 0 and 1. The probability distribution of the normal velocity component is directly proportional to cos $\theta$^13^.

In addition, for particle tracking, we used a GMRES solver as an iterative solver with a generalized-alpha time stepping method and a constant damping factor which is used as a nonlinear method in damped Newton iterations, using COMSOL Multiphysics 5.3.

To attain the amount of SDM adhered on the AAA lumen a specific variable was defined on each mesh element of AAA boundary surfaces. The variable was affected by all MBs that attached to the boundary elements. Whenever MB attached to the boundary element, the variable in that element was incremented by a source term which was set to one. Then the source term was divided by the area of the boundary element^12,13^.

### Forces exerting on the released particles

**Figure 2:**
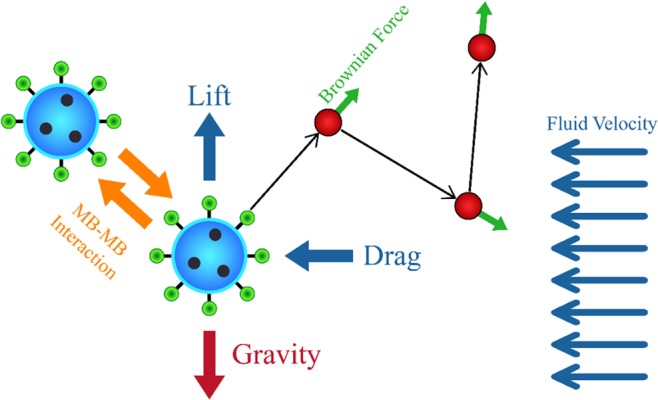
Forces which act on a MB in a fluid.

There are two types of forces that exerted on the MBs including the force from the external field on the MBs and the interaction force between MBs (See Fig. 2). The forces from external fields on the particles were calculated based on the finite element method. At each time step, the new location of MBs was queried based on the exerted external forces. After that, the forces from the interactivity of MBs were added to the total force. The position of MBs was then updated, and this process was repeated until the end of the simulation.

According to Newton’s second law for moving the MBs with mass ${m}_{p}$, can be written:

11 $$\frac{d}{dt}({m}_{p}{\bf{v}})={{\bf{F}}}_{{\rm{D}}}+{{\bf{F}}}_{{\rm{L}}}+{{\bf{F}}}_{{\rm{B}}}+{{\bf{F}}}_{{\rm{G}}}$$

where ${m}_{p}$ is the mass of the each particle, ${\bf{v}}$ is the velocity vector of the each particle, **F**_D_ is the drag force, **F**_L_ the lift force, **F**_B_ the Brownian force and **F**_G_ is the gravitational force acting on MBs.

#### Drag force

The drag force exerting on each particle has been defined as^51^:

12a $${{\bf{F}}}_{{\rm{D}}}=\left(\frac{1}{{\tau }_{p}}\right){m}_{p}({\bf{u}}-{\bf{v}}\,)$$

12b $${\tau }_{p}=\frac{4{\rho }_{p}{d}_{p}^{2}}{3{\eta }_{app}{C}_{D}{R}_{{e}_{r}}}$$

where ${\tau }_{p}$ is the particle relaxation time, ${\rho }_{p}$ indicates the particle density, ${d}_{p}$ is particle diameter, ${C}_{D}$ is drag coefficient and ${R}_{{e}_{r}}$ is the particle relative Reynolds number defined as follows:

13 $${R}_{{e}_{r}}=\frac{\rho ||{\bf{u}}-{\bf{v}}||{d}_{p}}{{\eta }_{app}}$$

To identify the drag coefficient, a range of standard drag correlations has been used as a piecewise function of the relative Reynolds number which has been reported by (Table 6)^52^. Since the maximum Reynolds number was 153.73 at the end of the simulation, the laminar flow assumption seems reasonable.

**Table 6:** The drag coefficient relations for different ranges of Reynolds number^52^.

Range	Correlation
${R}_{{e}_{r}}\le 0.01$	${{C}}_{{D}}=\frac{{\rm{24}}}{{{R}}_{{{e}}_{{r}}}}({\rm{1}}+\frac{{\rm{3}}}{{\rm{16}}}{{R}}_{{{e}}_{{r}}})$
$0.01\le {R}_{{e}_{r}}\le 20$	${{C}}_{{D}}=\frac{{\rm{24}}}{{{R}}_{{{e}}_{{r}}}}({\rm{1}}+{\rm{0.1315}}{{R}}_{{{e}}_{{r}}}^{{\rm{0.82}}-{\rm{0.05}}{w}})$
$20\le {R}_{{e}_{r}}\le 260$	${{C}}_{{D}}=\frac{{\rm{24}}}{{{R}}_{{{e}}_{{r}}}}\,({\rm{1}}+{\rm{0.1935}}{{R}}_{{{e}}_{{r}}}^{{\bf{0.6305}}})$

#### Lift force

When a spherical shape particle is immersed in viscous fluid flow, velocity discrepancy and consequently discrepancy in pressure between the upper and the lower side of the particle will generate a lift force which for a particle with small dimensions according to Saffman^53^ is as follows:

14 $${{\bf{F}}}_{{\rm{L}}}=-\,20.3\,{d}_{p}^{2}\,{{\bf{L}}}_{v}\sqrt{{\eta }_{app}\rho \frac{|{\bf{u}}-{\bf{v}}|}{|{{\bf{L}}}_{v}|}}$$

where ${{\bf{L}}}_{v}$ is follow:

15 $${{\bf{L}}}_{v}=({\bf{u}}-{\bf{v}})\times [\nabla \times ({\bf{u}}-{\bf{v}})]$$

Moreover, $\nabla \times ({\bf{u}}-{\bf{v}})$ is rotational flow field.

#### Brownian force

Fluid molecules which are in constant, rapid, random motion; are colliding with each other inside of the blood fluid. The procedure is called colliding. As a result of these colliding particles inside of the fluid will obtain a random motion which is called Brownian motion. This motion and the resultant exerting forces of this motion according to recent studies in particles with small scales, are considerable^13^. This force is defined by a Gaussian noise process which is as follows^13^:

16 $${{\bf{F}}}_{{\rm{B}}}={\boldsymbol{\zeta }}\sqrt{\frac{6\pi {K}_{B}{\eta }_{app}T{d}_{p}}{\varDelta t}}$$

In this equation ${K}_{B}$ is Boltzmann’s constant, $T$ is the absolute fluid temperature, Δ*t* is the magnitude of time step taken by the solver, and ${\boldsymbol{\zeta }}$ is a normally distributed random vector with a mean of zero and unit standard variation.

#### Gravitational force

The gravitational force on each particle flowing in a fluid has been given as^54^:

17 $${{\bf{F}}}_{{\rm{G}}}={m}_{p}{\bf{g}}\frac{({\rho }_{p}-\rho )}{{\rho }_{p}}$$

where ${\bf{g}}$ indicates the gravitational acceleration. Considering that in Pharmaceutical manufacturing process and imaging with the use of ultrasound MB, the patient is receiving medical attention while being lain down^13,21^. Therefor according to Supplemental Fig. S3, the gravitational force is in the direction of the y axis.

#### MB - MB interaction

To portray the interactions between MBs a Lennard-Jones potential is as follows^55^:

18 $${\bf{U}}({\bf{r}})=4\,\varepsilon \left[{\left(\frac{\sigma }{{\bf{r}}}\right)}^{12}-{\left(\frac{\sigma }{{\bf{r}}}\right)}^{6}\right]$$

where $\,\varepsilon$ is the interaction strength, $\sigma$ is the collision diameter, ${\bf{r}}$ is the distance between MBs, and ${{\bf{r}}}_{m}$ is the minimum distance as follows:

19 $${{\bf{r}}}_{m}={2}^{\frac{1}{6}}\sigma $$

As we know from the dynamics of particles, with the gradient of particle potential, the force between them is obtained as follows:

20 $${\bf{F}}=-\,\nabla {\bf{U}}$$

Therefore, the expression for the force on the ${i}^{th}$ particle becomes:

21 $${{\bf{F}}}_{{\rm{i}}}=\frac{24\varepsilon }{\sigma }\mathop{\sum }\limits_{j=1}^{N}\left[2{\left(\frac{\sigma }{|{{\bf{r}}}_{{\rm{i}}}-{{\bf{r}}}_{{\rm{j}}}|}\right)}^{13}-{\left(\frac{\sigma }{|{{\bf{r}}}_{{\rm{i}}}-{{\bf{r}}}_{{\rm{j}}}|}\right)}^{7}\right]\left(\frac{{{\bf{r}}}_{{\rm{i}}}-{{\bf{r}}}_{{\rm{j}}}}{|{{\bf{r}}}_{{\rm{i}}}-{{\bf{r}}}_{{\rm{j}}}|}\right)$$

For rigid surface MBs ${{\bf{r}}}_{m}={d}_{p}$ and According to Eq. (19), $\sigma =0.89\,({d}_{p})$. Also, the $\varepsilon =8.20\times {10}^{-22}$ was selected in this work and the cutoff length was equal to $55\,mm$^55^.

### MB Adhesive dynamic model

In the current study, it was assumed that the MBs surface is covered by P-selection aptamers (PSA). According to previous studies, these aptamers possess higher adhesion tendency compared to the other antibodies^33,56^. The ligand-receptor binding formulation was introduced by Decuzzi and Ferrari for the first time to calculate the probability of adhesion of particles^57^. Their model exerted force, $F$, and torque, $T$, on sphere particles under fluid flow, and wall shear stress defined the shape and size of the particle as^57^:

22a $$F=3\pi {d}_{p}l{\eta }_{app}S{F}^{s}$$

22b $$T=\frac{1}{2}\pi {d}_{p}^{3}\,{\eta }_{app}S{T}^{s}$$

where $l$ is the separation distance of the particles from the substrate and the ${F}^{s}$ and ${T}^{s}$ are two parameters depending on the particle aspect ratio ($\gamma =\frac{a}{b}$). In the study of spherical particles $\gamma =1$ the classical results in a research by Goldman and his colleagues^58^ were selected; $\,{F}^{s}=1.668$ and ${T}^{s}=0.944$. Especially, a probability of adhesion ${P}_{a}$ can be defined as the probability of having at least one close ligand–receptor bond. This probability of adhesion is directly related to the strength of adhesion that is; the larger is the greater is the adhesive strength of the particle to the endothelium. In the limit of a small surface density of receptors ${m}_{r}$ or ligands ${m}_{l}$ the probability of adhesion ${P}_{a}$ for spherical particles has the following form:

23a $${P}_{a}=\pi {r}_{0}^{2}{m}_{r}{m}_{l}{K}_{a}^{0}\times exp\left[-\frac{\beta {d}_{p}{\eta }_{app}S}{{K}_{B}T{r}_{0}^{2}{m}_{r}}\left[3\left(\frac{{d}_{p}}{2}+{\delta }_{eq}\right){F}^{s}+\frac{{d}_{p}^{2}}{{r}_{0}}{T}^{s}\right]\right]$$

23b $${r}_{0}^{2}={d}_{p}^{2}\left[\frac{1}{4}-{\left(\frac{1}{2}-\frac{({h}_{0}-{\delta }_{eq})}{{d}_{p}}\right)}^{2}\right]$$

where ${K}_{a}^{0}$ is the association constant at zero load of the ligand–receptor bound, $\beta$ is the reactive compliance which is usually in the order of angstrom, ${\delta }_{eq}$ is the equilibrium separation distance between the spheroidal particle and the vascular substrate, ${k}_{B}T$ is the Boltzmann thermal energy, ${h}_{0}$ is the maximum distance of the particle from the vascular wall at which a specific bond can take place, and ${r}_{0}$ is the radius of the circular section of the spheroid situated at a separation distance ${h}_{0}$ from the substrate.

Considering the Decuzzi and Ferrari equations, it can be shown that the strength of adhesion increased in sphere particles. Another study of Maeul *et al*. demonstrated that the optimisation of ultrasound contrast agents using a computational model of blood vessels leads to the improved selection of ligand and binding strength. In their works, they assumed that the surfaces of MBs have antibodies such as ligand and receptors located on the lumen wall (endothelial cells)^56^. The values of their parameters are presented in Table 7.

**Table 7:** Corresponding values of parameters used in the adhesive dynamics model for PSA: P-selectin^13^.

Parameter	Value
Intrinsic forward kinetic rate: ${K}_{f}^{0}({s}^{-1})$	2.33 × 10^5^
Intrinsic reverse kinetic rate: ${K}_{r}^{0}({s}^{-1})$	3.4 × 10^−5^
Equilibrium bond length: ${\delta }_{eq}(nm)$	50
Reactive compliance: $\beta (\AA )$	0.21
Boltzmann’s constant: ${K}_{B}({m}^{2}kg/{s}^{2}K)$	1.3806 × 10^−23^
Surface density of receptors: ${m}_{r}({m}^{-2})$	2.7 × 10^15^
Temperature: T(K)	310.15
Shear stress at the wall: ${\eta }_{app}S(Pa)$	Obtained from the FSI simulation

**Figure 3:**
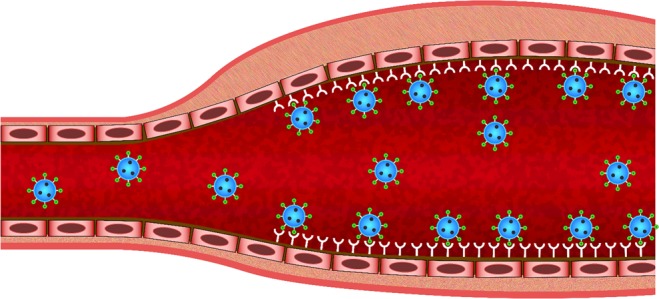
Schematic of targeted drug delivery of MBs using ligand-receptor binding to the AAA wall.

During targeted drug delivery by MBs, the drug is generally placed inside the MBs. By injecting these MBs into the artery and during their motion under blood circulation, some of the MBs may collide with the target wall. Now, if MBs possess antibody on their surface, they could adhere to the target wall. Upon, ultrasonic treatment, the MBs will burst and their drug content will be released. If the MBs are not adhered to the target wall, their released drug content may be far from the target and the MBs will be carried to the artery downstream by the blood circulation^7^ (See Fig. 3).

## Results

In the current study, partial differential equations were solved using COMSOL Multiphysics 5.3. For FSI simulation such as MUMPs algorithm, a PDF time-stepping technique and a tolerance termination procedure were also employed. The MBs transport was simulated using a solver which hired a backward Euler consistent initialization and the generalized-alpha time-stepping method^59–61^.

In this section, we first present the FSI simulation results, which include both negative and non-negative inlet velocities. It must be noted that vortex plays an important role in these velocities. Subsequently, the impact of MB size on the drug delivery efficiency was investigated in 4 types of MBs. For this purpose, the effect of SDM adhesion on AAA was addressed in the last cardiac cycle. For justification, the SDM adhesion was compared with other effective drug delivery parameters such as MBs kinetic energy (MKE) and MRT. In the third step, the effects of the blood flow velocity (both magnitude and the direction) and the vortex on the surface adhesion were investigated in the targeted delivery of drug-containing MBs. Then, the homogeneous distribution of MBs in the dichotomy part of the artery was examined. Finally, the amounts of forces applied to the microbubbles during targeted drug delivery are given.

### FSI results

Generally, to ensure that the AAA does not rupture, the maximum displacement of the its wall must be within a certain range. The maximum displacement $(2.056\,mm$) occurred in the second cardiac cycle at $t=2.03\,s$ (See Supplemental Fig. S4a). Also, the velocity magnitude at $t=2.03\,s$ is shown in two states of volume and slice in Supplemental Fig. S4b,c, respectively.

As mentioned earlier, simulations researches on AAA disease showed the dependence of the artery inlet velocity on the geometry and size of AAA which are different from one individual to another^34,62^. Our simulation presented a patient with a particular AAA geometry. However, the artery inlet blood flow velocity of this patient was not calculated. In the current study, an inlet velocity profile with maximum velocity and negative velocity (that in most researches were carried out on AAA) used the same procedure. To compare the results and investigate the impact of inlet velocity on the blood flow, the artery velocity profiles with non-negative velocities (in a cardiac cycle) and low maximum velocity were also included. Vortex generated inside the AAA can be used to investigate the effects of the artery inlet velocity. Supplemental Fig. S5 demonstrates the inlet velocity profile of the artery and the vortex magnitude diagram in the last cardiac cycle. It can be observed that both diagrams are similar; near the maximum inlet velocity, the amount of vorticity increased and reached to its pinnacle (See Supplemental Fig. S5a). This trend was also observed in the non-negative inlet velocity (See Supplemental Fig. S5b). Comparison of these two diagrams (Supplemental Fig. S5a,b) indicated that enhancement of the magnitude of inlet velocity will lead to an increment in the vorticity magnitude. So that for maximum magnitude of inlet velocity (44 $cm/s$), maximum vorticity magnitude $(52\,{s}^{-1})\,\,$occurred at $t=3\,s$ (See Supplemental Fig. S5a). Whereas for maximum inlet velocity (30 $cm/s$), maximum vorticity $(37\,{s}^{-1})$ was observed at $t=2.92\,$(See Supplemental Fig. S5b).

**Figure 4:**
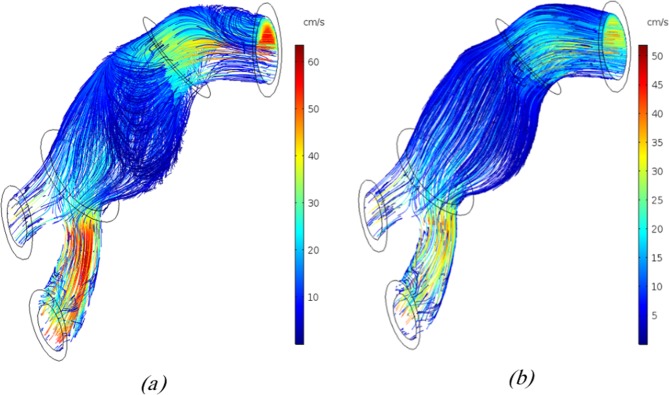
Flow streamlines coloured by velocity magnitude (m/s) for (**a**) negative inlet velocity and (**b**) non- negative inlet velocity at the time of maximum vorticity magnitude of blood flow (Generated by COMSOL Multiphysics 5.3, https://www.comsol.com).

Streamlines within the AAA are demonstrated in Fig. 4a,b for non-negative and negative inlet velocities at the moment of maximum vorticity, respectively. As Fig. 4 suggests, the vortices generated by the negative inlet velocity were higher than those produced by non-negative one; this could be due to higher blood flow toward the aneurysm internal wall for the higher artery inlet velocity. The reverse flow after contact with the inner surface of the artery generates vortices; the higher inlet velocities will enhance the magnitude of these vortices. Furthermore, according to recent studies, the AAA shape affects the vortex generation^34,63^. Moreover, fluid flow velocity was higher at outlet 2 when compared with the outlet 1; hence the AAA geometry can be considered as the primary cause of the mentioned observation.

One of the other differences in the artery inlet velocity profiles is the negative velocity during the cardiac cycle which can affect the fluid flow through the artery. The blood streamlines through the artery are depicted in Supplemental Fig. S6 when it reached its negative pinnacle in the third cardiac cycle. Chaotic blood flow is observable in the middle area of AAA which emanated from reverse fluid flow in the AAA (See Supplemental Fig. S6a). However, for non-negative inlet velocities, reverse fluid and chaotic flows were not observed when the velocity became zero (See Supplemental Fig. S6b). Reverse fluid flow within the AAA is expected to be useful in transporting drug carriers to the target wall during the cardiac cycle.

### The delivery efficiency concerning MBs type

In this section, the effects of different parameters on the MBs adhesion to the inner surface of the target will be investigated. First, different types of MBs with medium diameter were studied. For comparison and investigation of the SDM adhesion on the lumen AAA, the average MRT in the arterial blood domain and the average MKE were employed. Different percentages of blood Hct in the artery were also compared. Finally, for each type of MBs, the SDM adhesion was illustrated for three various diameters.

**Figure 5:**
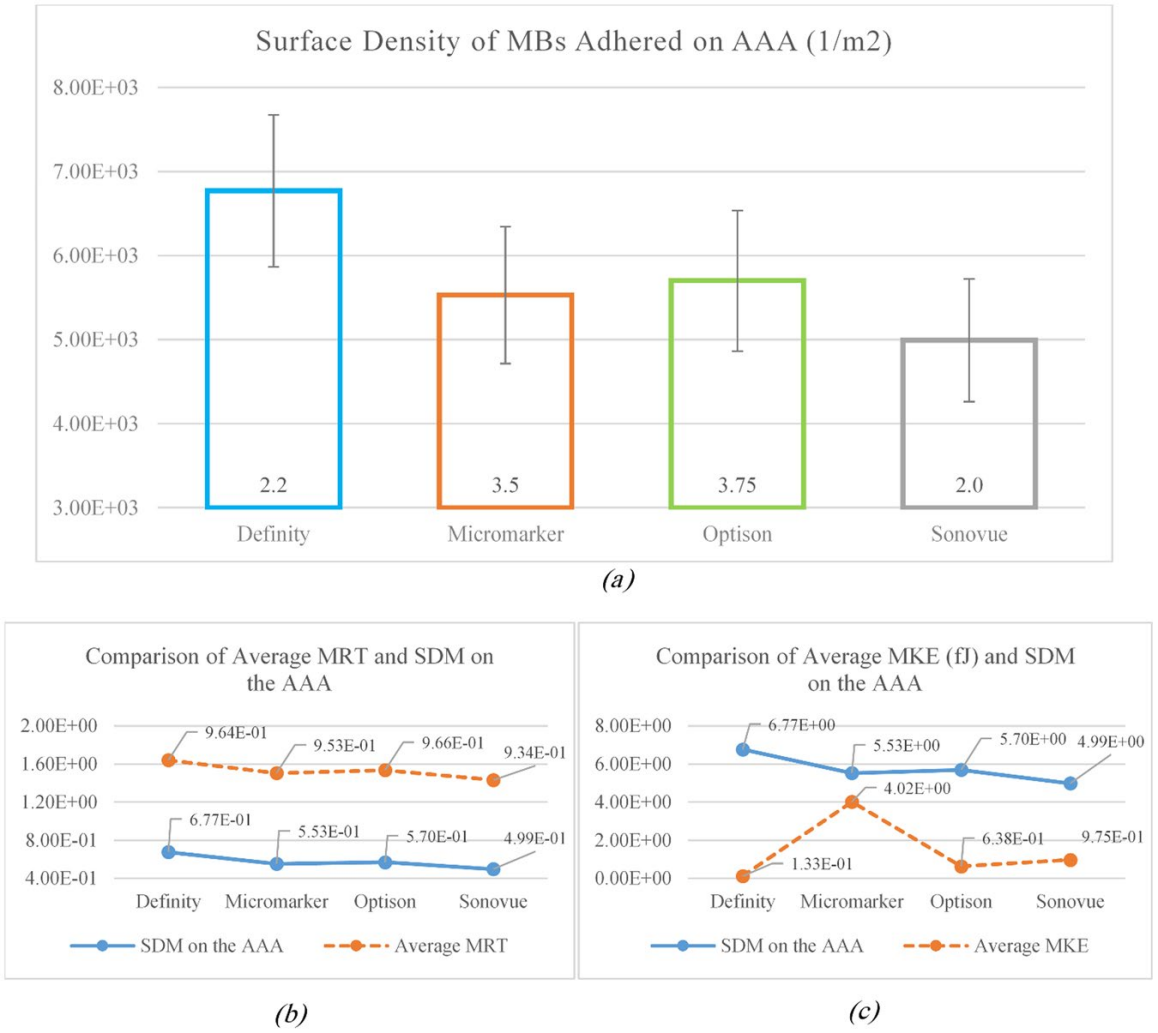
Variation of (**a**) the SDM adhered on AAA lumen for different types of MBs, (**b**) comparison of average MRT and SDM and (**c**) comparison of MKE and SDM.

According to Fig. 5a, the maximum SDM adhesion on the surface of AAA was observed in Definity MB with medium diameter while the minimum SDM adhesion was recorded in Sonovue MB. As the SDM adhesion of drug containers on the intended surface is directly related to the average MRT, MBs ligands will have more chances to bond with the receptors on the inner surface wall of the AAA by increase of the average MRT. This is evident in Fig. 5b, both the diagrams are the same; so, for MBs with higher residence time, the SDM adhesion was also greater and vice versa. Noteworthy, the average MKE exhibited a good relation with the SDM. Therefore, by enhancement of the average MKE inside the AAA, the probability of MBs adhesion to the inner surface wall will decrease. Figure 5c compares the average MKE and the SDM adhesion on the AAA where the SDM adhesion declined by growth of the average MKE.

As mentioned before, the mechanical properties of the blood can vary depending on the patient diseases and other parameters. A small change in the mechanical properties of the blood will alter the magnitude of the drag and lift forces exerting on the MBs. It can also induce changes in the viscous stress of the blood flow which can influence the density adhesion of drug-containing MBs. In this context, a comparison was made with three different Hct percentages (65, 45 and 25). The SDM adhesion to an AAA with diameter of 2.2 *μm* MBs is shown in Supplemental Fig. S7 for three different blood Hct percentages. As it can be seen, the MBs adhesion on the intended surface was lower for the blood possessing low Hct percentages as compared with the Hct percentage of 45 (healthy individual). Also, for the blood with high Hct percentage, the MBs adhesion on the inner wall will be dramatically decreased which is in line with the previous researches^12^.

**Figure 6:**
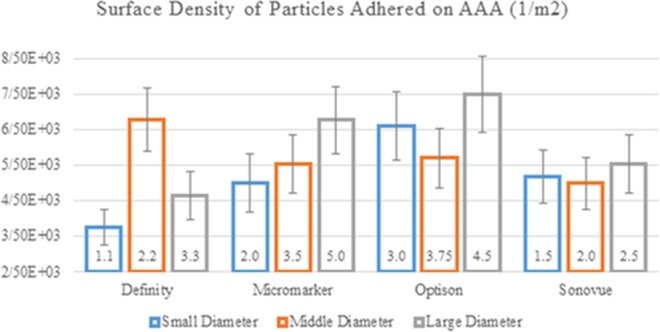
Variation of the SDM adhered to the AAA lumen for different types and sizes of MBs.

The size of MBs specimens was also compared. Three different diameters (minimum, maximum and average) were selected for each MB and their surface densities were compared as illustrated in Fig. 6. As Fig. 6 shows, the maximum SDM was detected in Definity, Micromarker, Optison, and Sonovue with diameters of 2.2, 5.0, 4.5 and 2.5 *μm*, respectively. Among all the studied samples, the maximum SDM was recorded in Optison with diameter of 4.5 *μm*.

### The delivery efficiency concerning MBs type for non-negative inlet velocity

**Figure 7:**
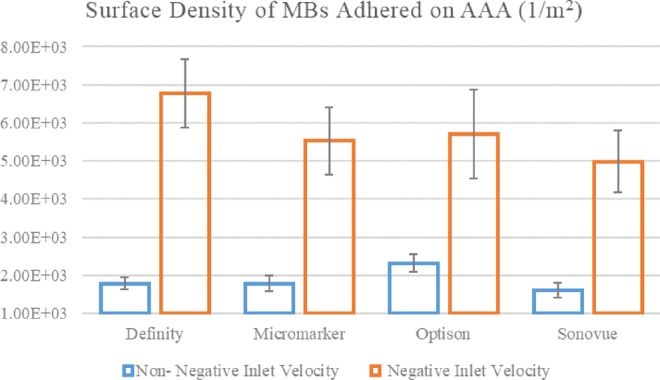
Variation of the SDM adhered on the AAA lumen for non-negative and negative inlet velocities.

As mentioned in section 3.1, inlet velocity of the artery can vary depending on different parameters and any changes in the artery inlet velocity can alter the blood flow through the artery. According to the recent studies, velocity variations in fluid flow can affect the bonds between the MBs ligands and the artery receptors^57^. In this section, the effects of the artery inlet velocity on the SDM adhesion will be addressed. The simulations were carried out by another inlet velocity profile that surpassed the recent velocity profiles in low velocity magnitude with no negative velocity in a cardiac cycle. The results showed that besides the reduction in the density adhesion, the patterns of minimum and maximum MBs density were changed compared to the previous state. According to Fig. 7, the maximum SDM adhesion for non-negative inlet velocity occurred for Optison at diameter of 3.75 *μm*; while the minimum SDM was observed in Sonovue with diameter of 2.5 *μm*. This observation was achieved by comparing the average MRT and the SDM adhesion diagrams for non-negative velocities (See Supplemental Fig. S8).

### The uniformity of MBs distribution

As was mentioned before, AAA refers to an inflammation with different geometries and dimensions in the abdominal part of the artery. In some cases, this inflammation occurred in the dichotomy of the terminal part of the artery (See Supplemental Fig. S9). Generally, injection of drug-containing carriers for patients with an aneurysm in the abdominal and/or dichotomy parts of the artery is performed from the beginning of the abdominal aortic. Therefore the prediction of the amount of the MBs distribution in the dichotomy part is of crucial importance. The homogeneity index indicates the transmission probability of MBs released to the two outlets of the aortic artery. The uniformity of MBs distribution in the AAA arterial network was evaluated in this investigation; in which four MBs types were addressed considering the average diameter for both negative and non-negative inlet velocities. Supplemental Fig. S10a demonstrates the homogeneity with consideration of outlet 2 to outlet 1 ratio. As it can be observed, Definity with 2.2 *μm* diameter exhibited the maximum homogeneity while the lowest value was observed in Micromarker with 3.5 *μm* diameter. In the case of non-negative inlet velocities Supplemental Fig. S10b, the maximum and minimum values were detected in Optison with diameter of 3.75 *μm* and Definity with diameter of 2.2 *μm*, respectively. Comparison of these two plots indicated the significant difference in homogeneity index for these two velocities; where the non-negative inlet velocity resulted in higher order homogeneity. This means that for non-negative inlet velocities, more number of MBs exist at outlet 2 compared to the outlet 1.

### The forces effect on the targeted drug delivery

Drug-containing MBs inside of the artery experience various forces such as drag, lift, Brownian and MB-MB interaction forces as well as other body forces including the gravitational force. Any of these forces can affect the SDM adhesion. Therefore, investigation of the mentioned forces can enhance our perception on the targeted drug delivery through the artery. The role of these forces in targeted drug delivery is investigated in this section with the emphasis on previous studies.

#### Drag and lift forces

Drag forces on the different types of MBs were calculated for three various diameters at negative and non-negative inlet velocities. Supplemental Fig. S11 demonstrates the drag forces on MBs based on their diameters for negative and non-negative velocities. It can be observed that the drag forces linearly changed by diameter increment. Neglecting the chaotic blood flow and parameters such as Reynolds number in drag coefficient term in Table 6, and substituting the achieved relation for drag coefficient in Eq. (12), it can be seen that the drag force has a linear relationship with MBs diameters. However, the drag force on MBs moving through blood flow depends on various parameters such as shape and size of MBs, their velocity, blood flow through the artery, Reynolds number and drag coefficient. On the other hand, the drag coefficient is also dependent on the MBs shape and orientation relative to the flow, Reynolds number, Mach number, and irregular level. Therefore, the drag force and reverse flow are unpredictable for flows with high vortex and irregular levels^64^. Comparing the drag forces for negative and non-negative inlet velocities, it can be concluded that the drag force exerted on MBs is higher in negative inlet velocities compared to the non-negative ones (See Supplemental Fig. S11).

**Figure 8:**
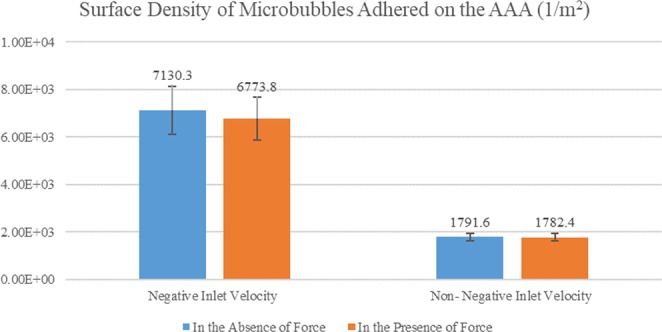
Variation of the SDM adhesion on AAA lumen to check the effect of the lift force.

Lift force is originated from the particles circulation. This circulation can be due to velocity gradient or other parameters such as MBs contact and rebound from a surface^64^. In recent studies on targeted pharmaceutical products, solid particles are employed as the drug carrier. The lift force has been neglected due to its fiddling nature^12,13^. However, studies have shown that MBs undergo a transfiguration due to high lift^64^. Therefore, the lift force was considered in this study. According to the results, although in comparison with drag force, the lift force is fiddling, but it can’t be neglected in the simulation process. Supplemental Fig. S12 shows the lift force plot versus the diameter of MBs in negative and non-negative inlet velocities. As it can be seen, the lift force changes by MBs diameter in a quadratic manner as justified by Eq. (14). According to Eq. (14), the lift force increases by enhancement of the fluid flow velocity. We can conclude this fact by comparing the lift forces in Supplemental Fig. S12a,b. For better understanding of the effects of lift forces on the simulation results, the SDM adhesion to the wall with 5 *μm* diameter was investigated with and without the lift force inclusion for both negative and non-negative inlet velocities (See Fig. 8). The influence of lift force on negative inlet velocity could be attributed to the high vortex generation and fluid flow circulation due to the reverse flow fully described in section 3.1.

#### Brownian, gravitational and MB-MB interaction force

**Figure 9:**
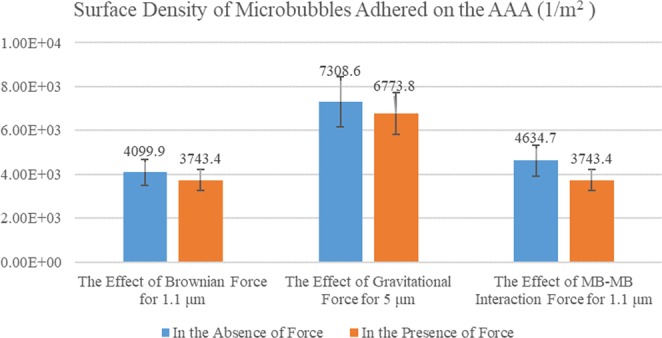
Variation of the SDM adhesion on AAA lumen to check the effect of the Brownian, gravitational and MB-MB interaction forces.

In this study, the Brownian force was also considered in the simulation process. Recent studies on the effects of Brownian forces on solid particles have shown that this force is fiddling for particles with the micrometre scale. This force is however not negligible in nanometer realm^12^. In this study, the effect of this force was investigated on the SDM adhesion. Figure 9 shows the SDM adhesion in two states: with and without considering Brownian forces. To demonstrate the effect of the Brownian forces, minimum diameter (i.e. 1.1 *μm*) was addressed. According to Fig. 9, the effect of Brownian force on the SDM adhesion was lower than 10% which is negligible in comparison with recent studies on the solid nanoscale particles^13^.

For demonstrating the effect of the gravitational force, maximum diameter (i.e. 5 *μm*) were used as it had high gravitational effect. Based on Fig. 9, the effect of the gravitational force on MBs is low (∼7.3%). Our recent studies on solid drug-containing particles indicated that the gravitational force has a positive impact on surface adhesion^13^. In this study, the gravitational force exhibited a negative impact on MBs. The main reason could be the density of the drug-containing particles. Unlike the solid particles, the density of MBs is lower than the blood density. Hence, according to Eq. (17), the gravitational force will negatively affect the SDM adhesion.

According to Eq. (21), the MB-MB interaction force is more significant in the small diameter of the MBs. Therefore, we used the smallest MB diameter (1.1 *μm* to check the effect of MB-MB interaction force on the SDM adhesion. As shown in Fig. 9, MB-MB interaction force had ∼19.2% impact the SDM adhesion on the lumen AAA. This impact is more than the Brownian and gravitational forces highlighting the importance of this force in the targeted drug delivery using MBs.

## Discussion

In this study, the adhesion of the drug-carrying MBs to AAA wall was investigated. FSI simulation results indicated that the maximum displacement of the artery wall in one cardiac cycle under blood pressure is 2.056. This is in line with the results obtained by the resent FSI simulations concerning AAA^62,65^. Our FSI results revealed higher vortex values for the negative inlet velocities when compared with the non-negative ones. Simulation of MBs motion within AAA showed the highest SDM for Definity MBs with diameter of 2.2 *μm*. The SDM adhesion on the intended surface depends on various parameters such as mechanical properties of MBs (surface density and dynamic viscosity), size and the shape of the MBs and AAA, mechanical properties of the blood and artery wall and the blood flow in the artery which itself depends on the inlet velocity and outlet pressure. Therefore the process of adhesion of the MBs is unpredictable and nonlinear^12,13,66^. However, their well-defined relationship can be understood by comparison of the mean MRT and mean MKE of MBs (shown in Fig. 5b,c). SDM adhesion is varied by changing the mechanical properties of the blood from healthy to diabetic and anemia. In these patients, SDM adhesion to AAA was lower compared to this parameter in healthy individuals. These observations can be explained by the fact that the Hct percentage of a healthy individual depends on the nonlinearity of the blood viscosity. Moreover, the Hct percentage depends on the magnitude of non-shear terms of the viscous stress tensor and the shear stress at the lumen surface^12^. These results highlight the key role of the blood mechanical properties on SDM adhesion to AAA. SDM adhesion was determined and compared for all types of MBs with three different diameters (minimum, moderate and maximum). This comparison can be a good criterion for designing MBs by specimen and size which can be used for similar diseases.

**Figure 10:**
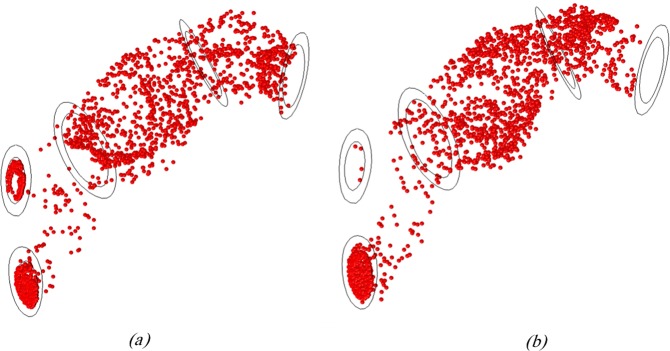
MBs dispersion in AAA domain for (**a**) negative inlet velocity and (**b**) non- negative inlet velocity at t = 3.6 s (Generated by COMSOL Multiphysics 5.3, https://www.comsol.com).

Regarding section 3.1, a high inlet velocity may cause vortex inside the AAA which in turn can increase the average MRT and consequently enhance the SDM adhesion to the inner wall surface of AAA. The reverse blood flow is another essential factor in the SDM adhesion to the artery as the artery inlet velocity becomes negative at the end of the systolic and the beginning of the diastolic phases. The reverse blood flow to the internal wall of the artery can increase the average MRT which eventually enhances the density adhesion of MBs on the AAA surface. Results revealed that for non-negative inlet velocities, the highest adhesion can be observed for the Optison with a diameter of 3.75 μm. However, for negative inlet velocity, this type of MB does not have the maximum adhesion on the aneurysm wall, indicating a significant influence of the velocity input properties on SDM adhesion to the artery wall (See Fig. 7). For better understanding this, the Poincare map of the Definity MBs distribution (with diameter of 2.2 *μm* at $t=3.6\,s$ was plotted for two negative and non-negative inlet velocities in Supplemental Fig. S13. Comparison of these figures showed that the MBs accumulation close to AAA wall surface was higher for MBs undergoing negative inlet velocity. Furthermore, the density of MBs at the middle side of the AAA was higher in negative inlet velocities. This matter was better demonstrated with MBs dispersion graphics in Fig. 10 which showed MBs dispersion for both negative and non-negative inlet velocities at the last cycle. As Fig. 10 demonstrates, more MBs were adhered to AAA for negative inlet velocity. Results also suggest that for non-negative inlet velocities, more MBs can be found in outlet 2 (compared to outlet 1). This observation is justified by producing a chaotic current in AAA with negative inlet velocity, which leads to a higher number of MBs exiting from outlet 1 (compared to non-negative inlet velocity). For non-negative inlet velocities, this phenomenon does not occur (See Fig. 10). The reason for higher number of MBs exiting outlet 2 for both negative and non-negative inlet velocities could be searched in the AAA and the dichotomy part geometries.

In this study, four types of MBs with different mechanical properties were used. Also according to Table 5, each of them is made of different diameters. A summary of the maximum amount of SDM to the inner wall of the AAA for two patients with negative and non-negative inlet blood flow to the aortic artery based on the type and size of MBs is presented in Table 8. For better drug delivery performance to AAA by MBs base on their size, it is recommended to use diameters of 2.2, 5, 4.5, and 3.75 *μm* for Definity, Micromarker, Optison, and Sonovue MBs, respectively. It is also advisable to select Optison with a diameter of 4.5 μm as the type of MBs for drug delivery to AAA with the highest SDM. For patients with non-negative inlet velocity, Optison performs better in drug delivery to AAA than other MBs.

**Table 8:** Values of the surface density of MBs adhered base on the type and size of MBs for negative and non-negative Inlet velocity profile with PSA: P-selectin.

Inlet velocity profile	*Microbubble*	Diameter [*μm*] (At maximum SDM adhered)	Surface density of MBs adhered
With negative magnitude	Definity	2.2	6.77 × 10^3^
	Micromarker	5.0	6.77 × 10^3^
	^*^Optison	4.5	7.49 × 10^3^
	Sonovue	2.5	5.53 × 10^3^
With non-negative magnitude	^*^Optison	3.75	2.3 × 10^3^

Investigation of the forces applied to the MBs during intra-artery drug delivery can improve our understanding of the effect of each of these forces on the SDM adhesion. According to the simulation results, the drag force applied on various MB types showed a linear increase with the diameter enlargement for non-negative inlet velocities. In the case of negative inlet velocities, however, it is not predictable due to nonlinearity of the problem, turbulence, and high vortex values (See Supplemental Fig. S11). Moreover, the drag force applied to MB is higher for the negative inlet velocities when compared with the cases involving non-negative velocities. As the diameter increases, the lift force exerted on MB increases quadratically. Comparison of the MBs’ adhesion to AAA considering and ignoring lifting forces showed that this force has no significant impact on the adhesion; but it significantly was different in two the cases of negative and non-negative inlet velocities (See Fig. 8). According to the results, the influence of Brownian forces and gravity on SDM adhesion of drug-carrying MBs to AAA wall is significant.

There are several limitations in this study. First, despite the shape diameter diversity in AAA, we only simulated the geometry with 40 mm diameter. The shape of the aneurysm can affect the blood flow through the artery, and as a result, the SDM adhesion on aneurysm surface. This matter should be investigated in future studies. Second, the effects of ultrasound force on MBs were neglected. Acoustic waves have been widely employed in targeted drug delivery. For instance, they can be used to guide the MBs to the target or burst the MBs at specific pressure and frequency. MB-assisted imaging or targeted drug delivery can be achieved by bursting the MBs after their adhesion to the target wall. In this study, the effect of acoustic forces on the MB adhesion to the target wall was neglected due to their small impact on the MBs motion^7,67,68^. The current study addressed the SDM adhesion on the intended surface using the MBs ligands and AAA receptors. Adhesion of different MBs on the intended surface was compared. Furthermore, the effects of the blood flow through AAA on the drug delivery to the intended target were also studied. However, according to the previous studies, the ultrasound wave force will burst the MBs by oscillating them to their critical point known as the resonance point; where the effect of linear displacement is low^68^. Yet, the effects of acoustic waves on the SDM adhesion to the intended surface should be investigated in future studies. The particle adhesive dynamics model used in this study was the PSA type. Recent studies have presented several models for particles adhesion with the receptor-ligand bond which can be employed for better investigation of the SDM adhesion^33^. Furthermore, in this simulation, 500 MBs were used at the beginning of each cardiac cycle. The number of MBs entering artery can be optimised according to the SDM adhesion to the intended surface, the number of MBs exiting in dichotomy and economic and health condition of the patient. Recently, a research was conducted on electromagnetic MBs in which the drugs can be delivered to the target by electromagnetic forces^69^. Application of an electromagnetic field can further enhance the MBs adhesion which can be the topic of the future researches.

## Conclusion

Simulation can be a valuable tool for investigating biological phenomena^12,13,33,70^. In this study, we employed simulation for studying the targeted drug delivery of MBs to suppress the AAA growth. In summary, we have shown that one of the commercially- approved MBs (Optison) with a diameter of 4.5 μm performs better than the other MBs in targeted drug delivery to the inner wall of the AAA. Our results showed that for patients with diabetes and anemia whose blood mechanical properties are variable in comparison with healthy individuals, the performance of targeted drug delivery using MB for the AAA would be decreased. Also, in patients with non-negative inlet blood flow velocity, the amount of SDM in the inner wall of AAA noticeably decreases, which reduces the effect of drug delivery with MBs to the AAA.

## Supplementary information

Supplementary information.
