# Supplementary material for: Targeted Drug Delivery of Microbubble to Arrest Abdominal Aortic Aneurysm Development: A Simulation Study Towards Optimized Microbubble Design
Source: Sci Rep. 2020 Mar 25;10:5393. doi: 10.1038/s41598-020-62410-3 (PMC7096410; doi:10.1038/s41598-020-62410-3)
Supplement: Supplementary file 1 — Supplementary information. [file 41598_2020_62410_MOESM1_ESM.docx]

**Targeted Drug Delivery of Microbubble to Arrest Abdominal Aortic Aneurysm Development: A Simulation Study Towards Optimized Microbubble Design**

Amir Shamloo^1, *^, Sina Ebrahimi^1^, Ali Amani^1^, Famida Fallah^1^

^1^School of Mechanical Engineering, Sharif University of Technology, Tehran, IRAN

*^*^Corresponding Author: Dr. A. Shamloo,* School of Mechanical Engineering, Sharif University of Technology,
Azadi Ave., Tehran, IRAN, Tel: 98-21-66165691, Fax: 98-21-66165599, email: [*shamloo@sharif.edu*](mailto:shamloo@sharif.edu)

### Supplemental Figures

*
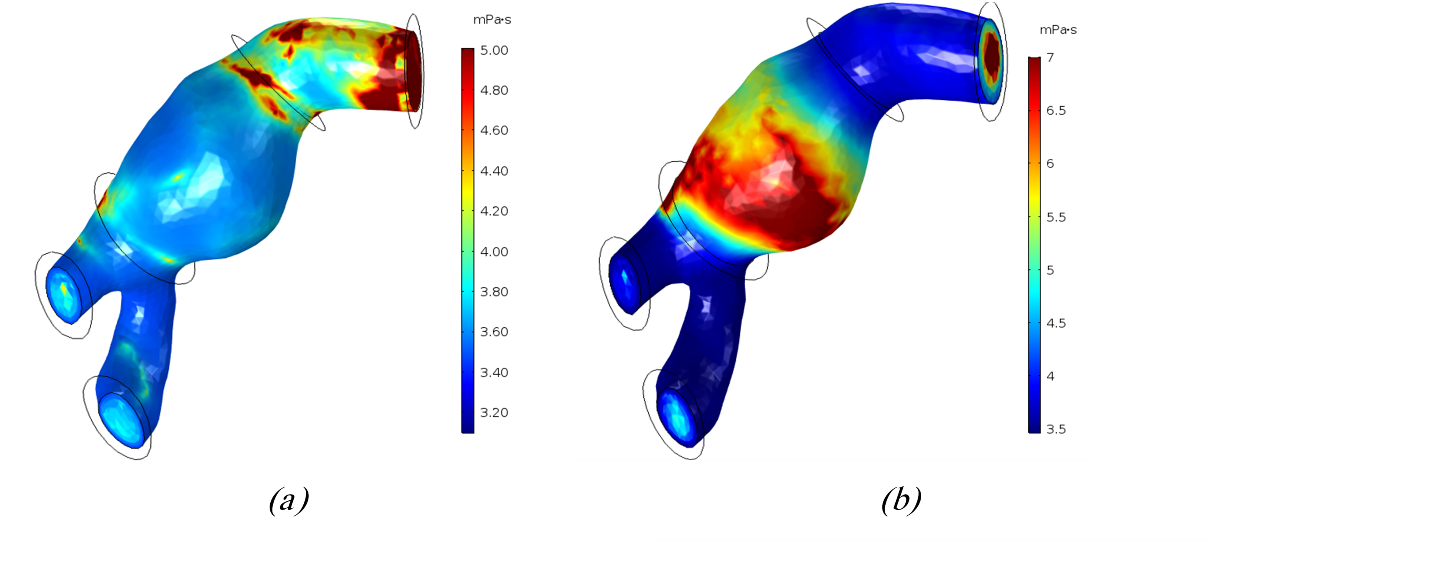
*

***Supplemental Figure S1*** *Field of apparent blood dynamic viscosity for (a) negative inlet velocity at time 2.69 s and (b) non- negative inlet velocity at time 3.14 s in the last cardiac cycle (Generated by COMSOL Multiphysics 5.3, https://www.comsol.com)*

*
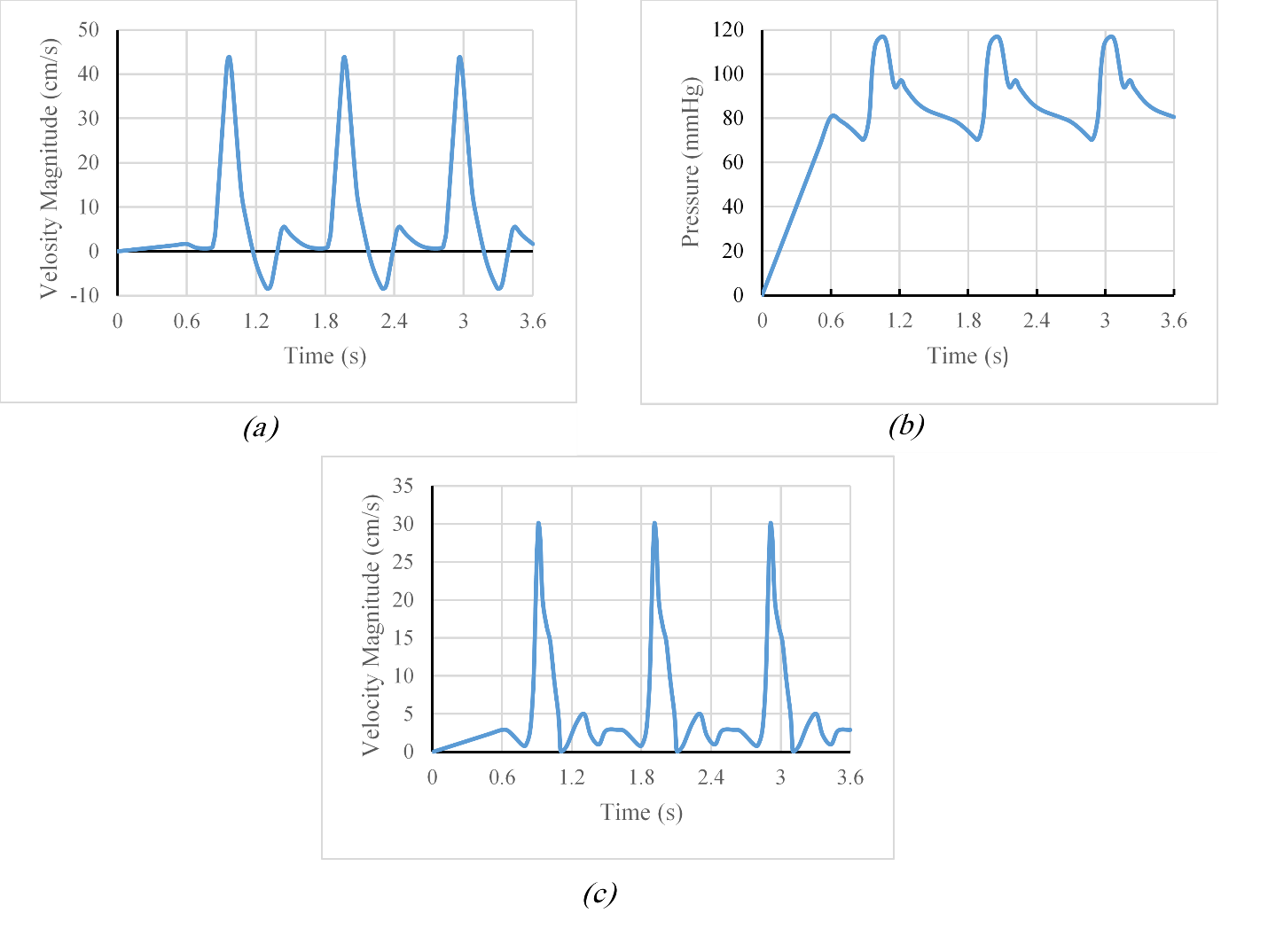
*

***Supplemental Figure S2*** *Inlet fluid negative velocity (a), pressure (b) and Inlet fluid non-negative velocity (c) waveforms indicating the peak systolic conditions of the cardiac cycle*


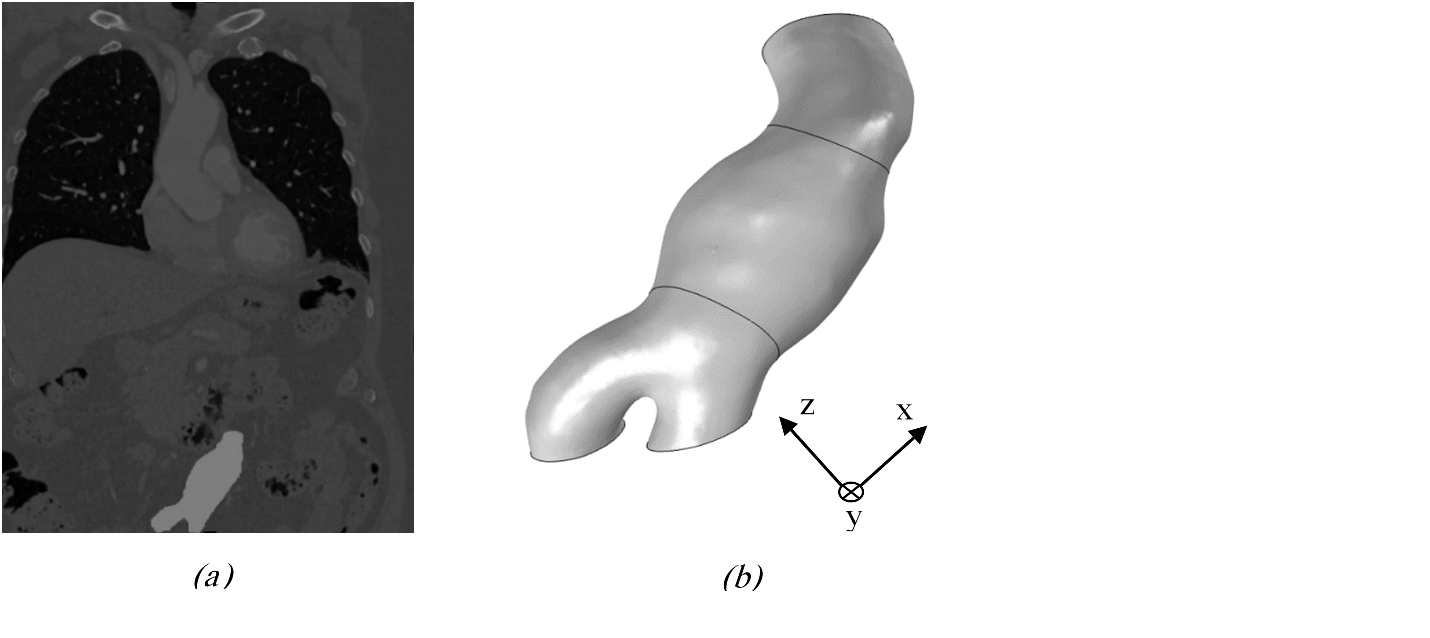


***Supplemental Figure S3*** *(a) The CT-scan image of the AAA in level condition and (b) the direction of y-axis accordingly selected for the gravitational force vector (Generated partly by COMSOL Multiphysics 5.3,* [*https://www.comsol.com*](https://www.comsol.com)*)*

***
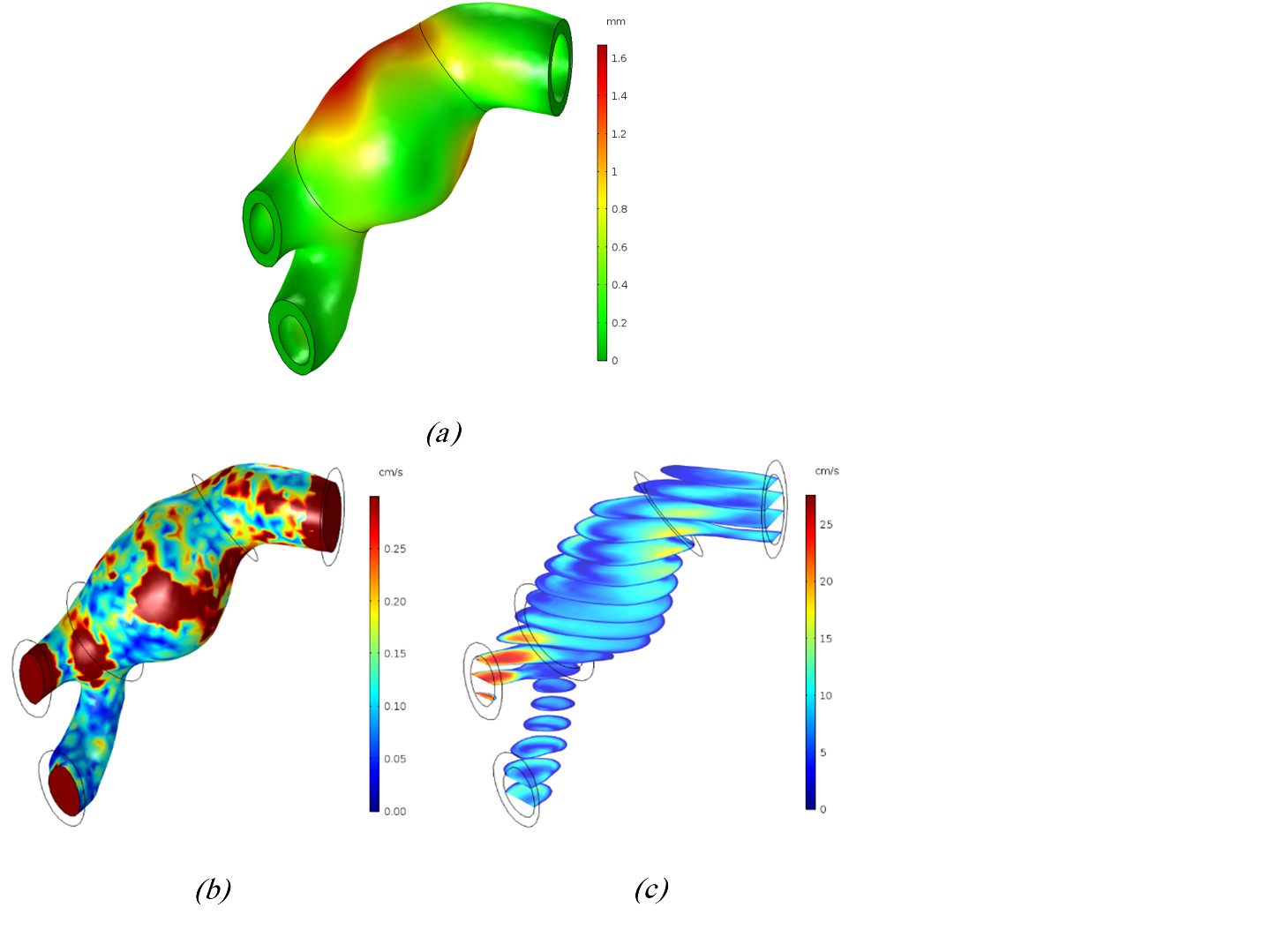
***

***Supplemental Figure S4*** *(a) Graphical variation of AAA wall total displacement and velocity magnitude in (b) volume and (c) slice state, around the second cardiac cycle (Generated by COMSOL Multiphysics 5.3, https://www.comsol.com)*

*
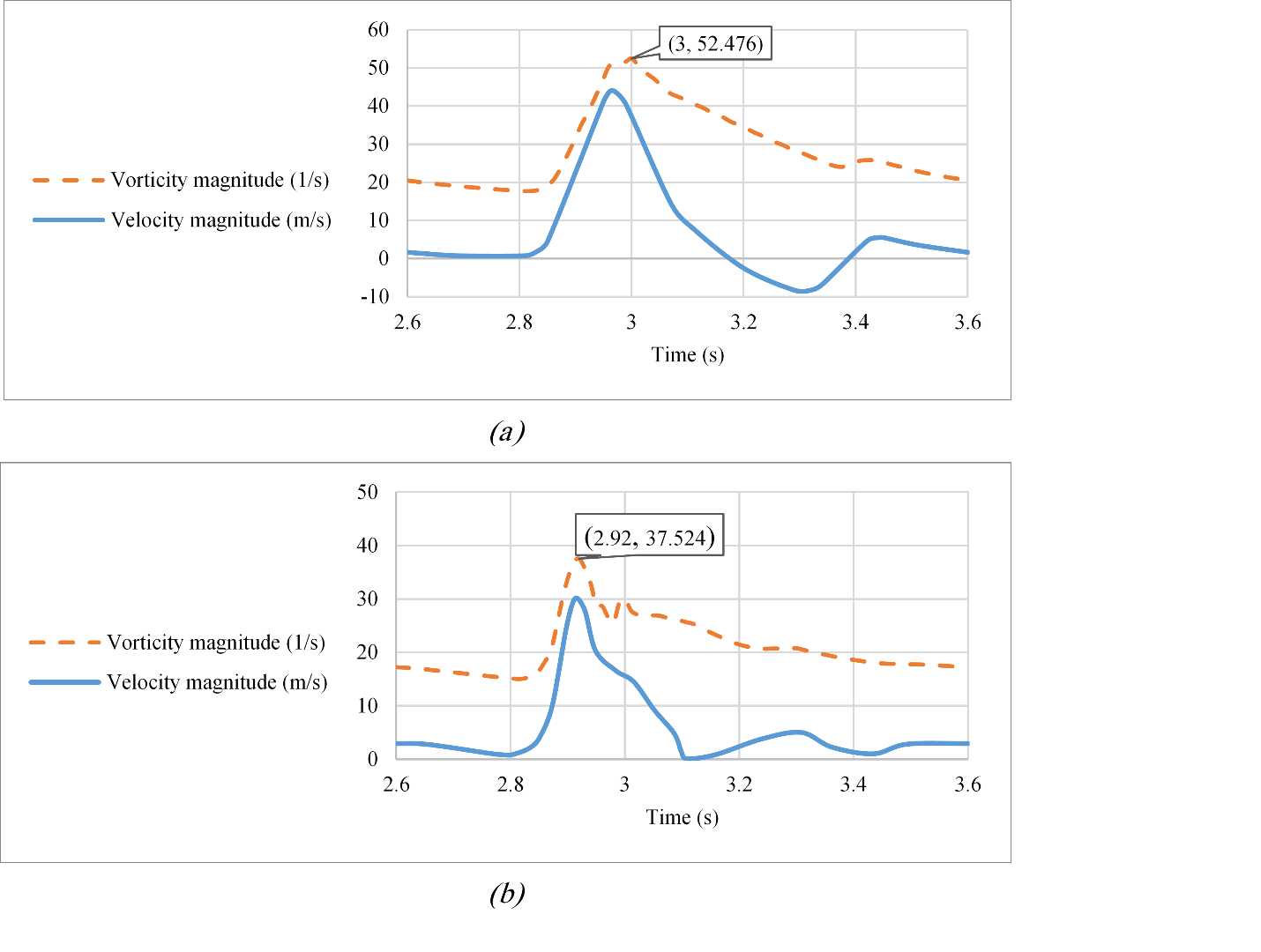
*

***Supplemental Figure S5*** *Comparison of velocity magnitude and vorticity magnitude of blood flow for (a) negative inlet velocity and (b) non- negative inlet velocity
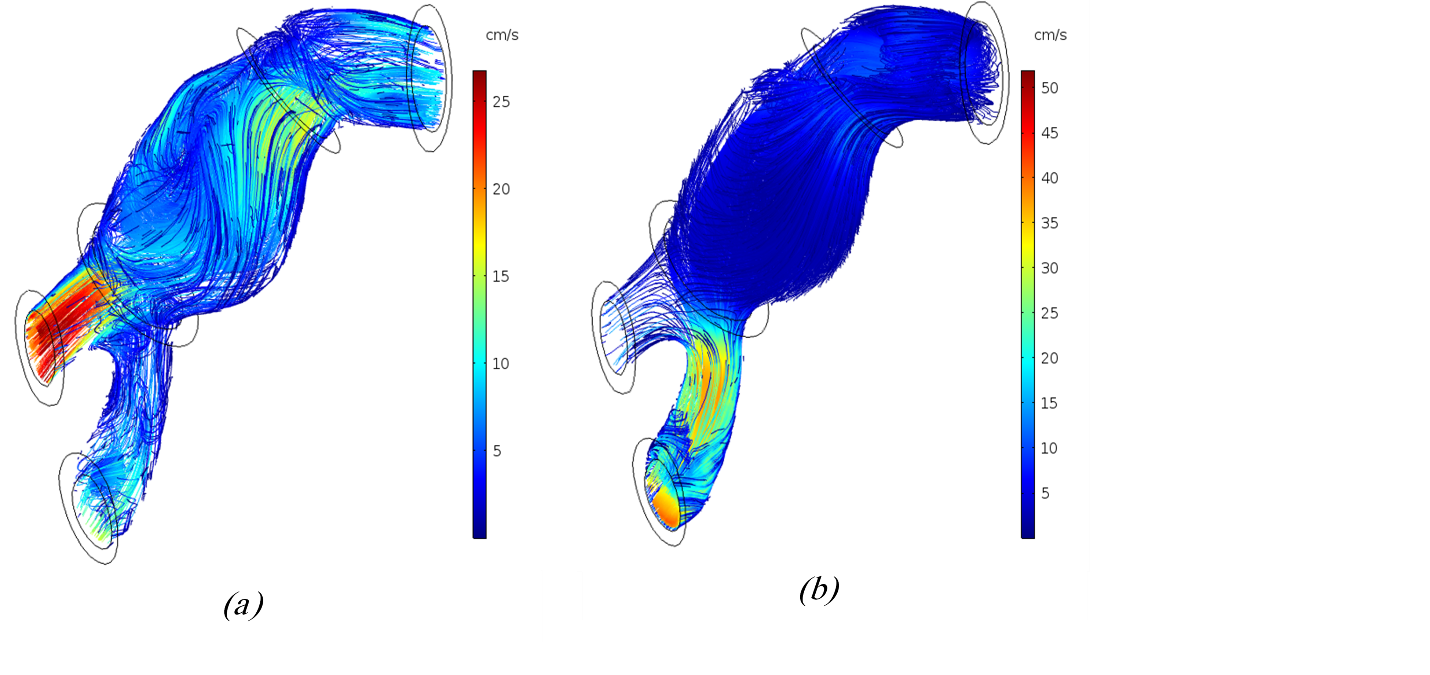
*

***Supplemental Figure S6*** *Flow streamlines coloured by velocity magnitude (m/s) for (a) negative inlet velocity and (b) non- negative inlet velocity at the time of minimum velocity magnitude of blood flow (Generated by COMSOL Multiphysics 5.3, https://www.comsol.com)*

*

*

***Supplemental Figure S7*** *Variation of the SDM adhered on AAA lumen in different blood Hct percentages*





***Supplemental Figure S8*** *Variation of average MRT through artery for non-negative inlet velocity*

*
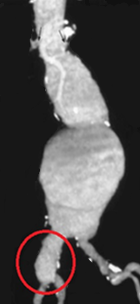
*

###### **Supplemental Figure S9** Distribution of aneurysm in dichotomy part of the aorta

***
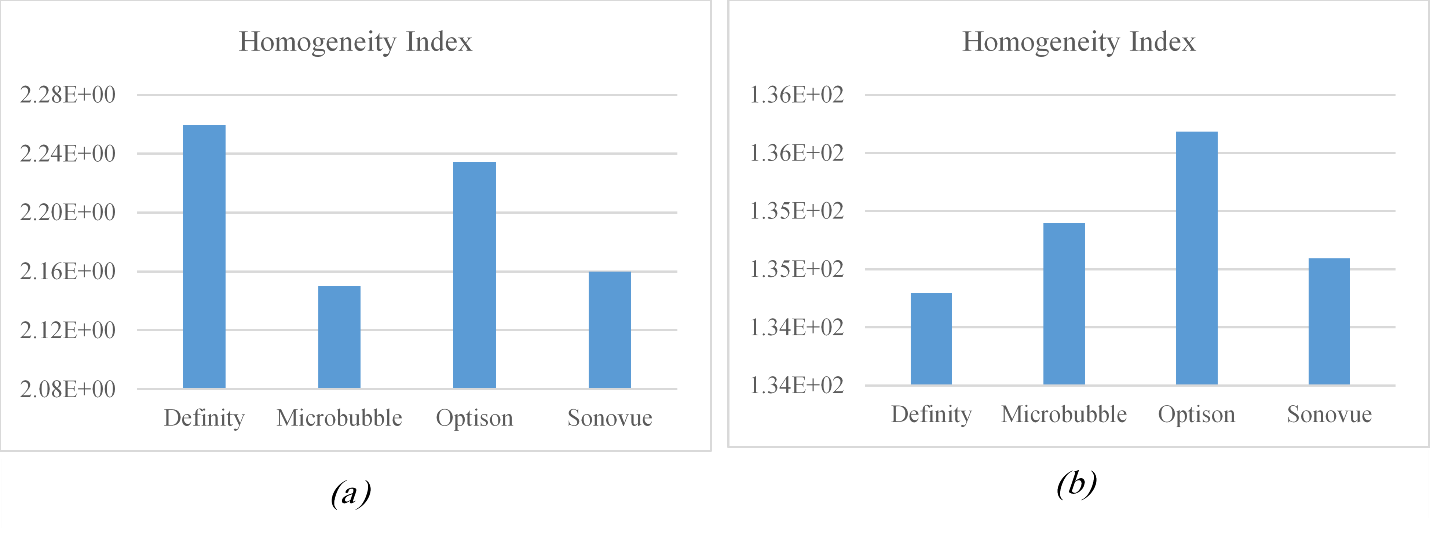
 Supplemental Figure S10*** *The uniformity of MBs distribution evaluated by the homogeneity index for (a) negative inlet velocity and (b) non- negative inlet velocity*

*
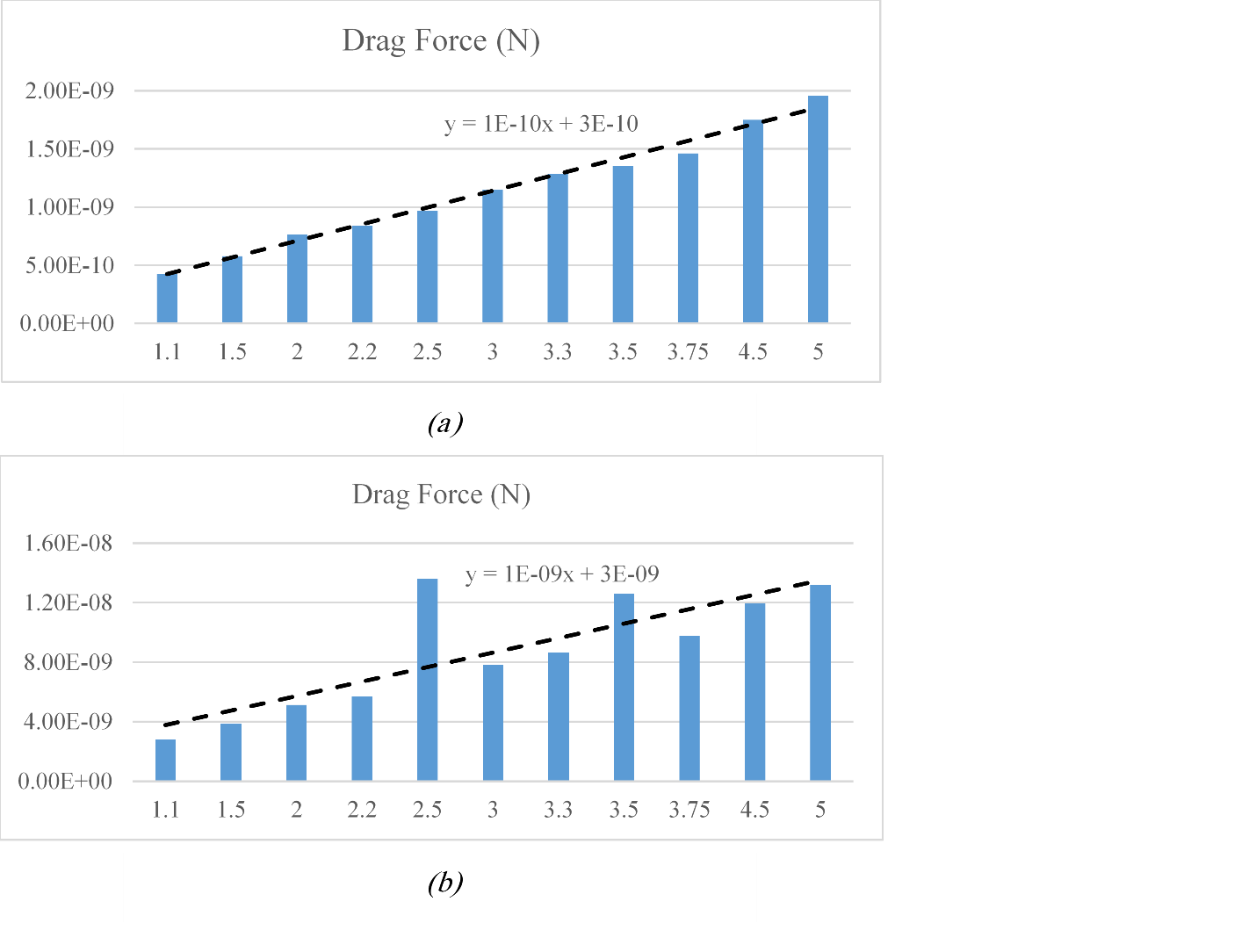
*

***Supplemental Figure S11*** *Variation of drag force of MBs for (a) non- negative inlet velocity and (b) negative inlet velocity*

*
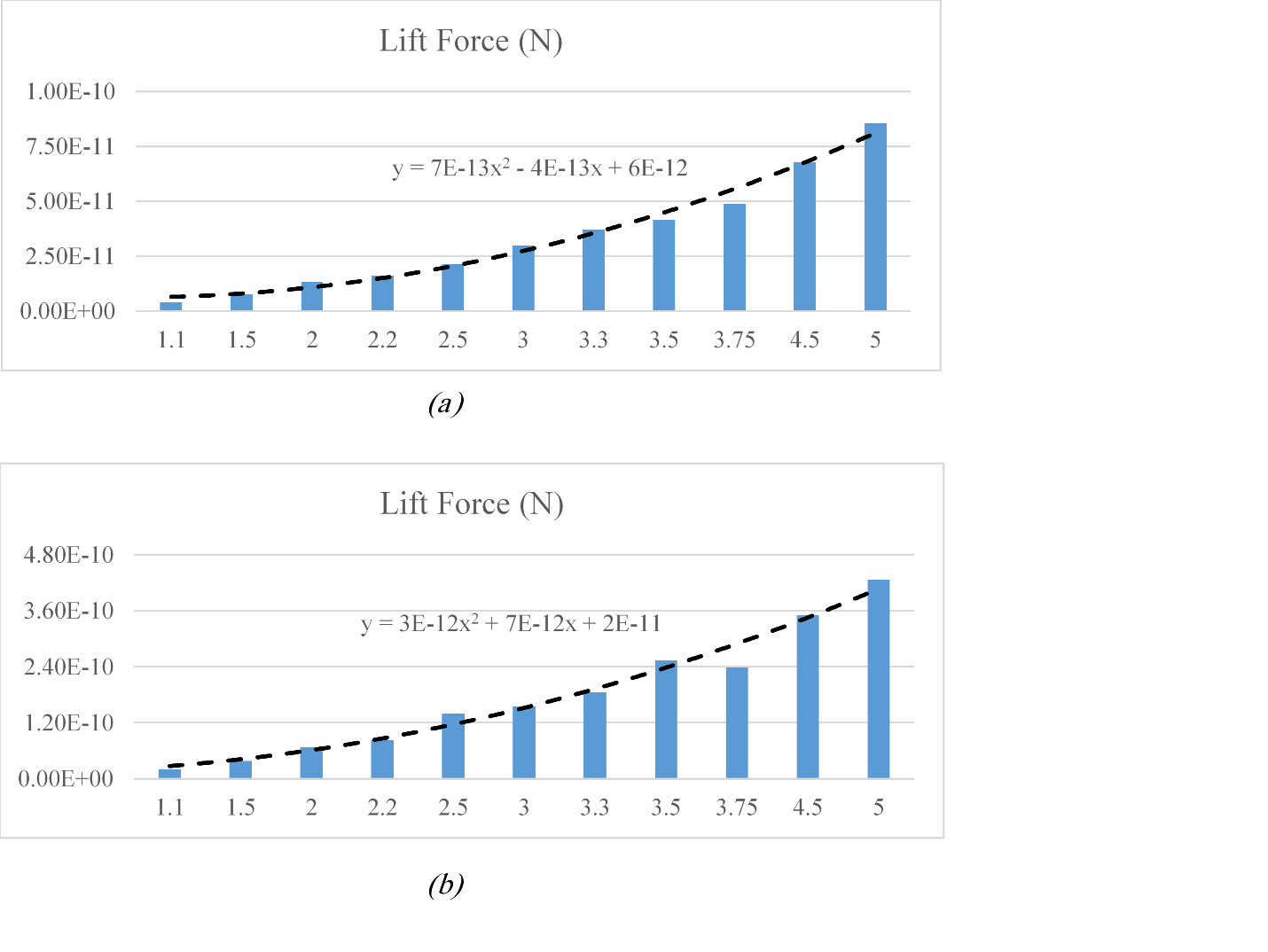
*

***Supplemental Figure S12*** *Variation of lift force of MBs for (a) non- negative inlet velocity and (b) negative inlet velocity*

*
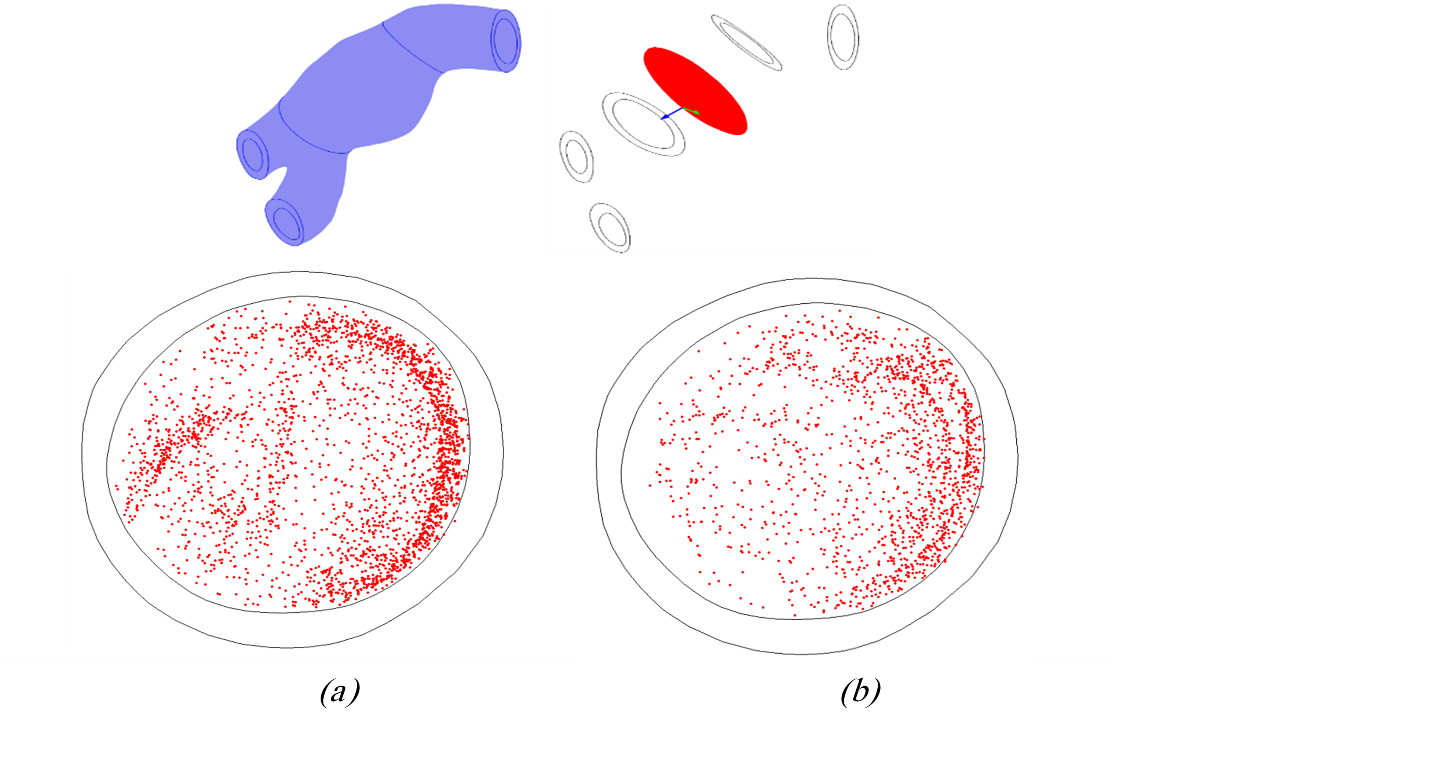
*

***Supplemental Figure S13*** *Poincare maps of the distribution of MBs for (a) negative inlet velocity and (b) non- negative inlet velocity at t=3.6 s (Generated by COMSOL Multiphysics 5.3, https://www.comsol.com)*
